# The complete mitogenome of *Curculiochinensis* (Chevrolat, 1878) (Coleoptera: Curculionidae: Curculioninae)

**DOI:** 10.3897/BDJ.9.e69196

**Published:** 2021-10-25

**Authors:** Kai Hu, Nian-nian Zhang, Zai-Hua Yang

**Affiliations:** 1 Guizhou Academy of Forestry, Guiyang, China Guizhou Academy of Forestry Guiyang China

**Keywords:** mitochondrial genome, camellia weevil, phylogenetic analysis, secondary structure

## Abstract

The mitogenome of *Curculiochinensis* (Chevrolat, 1878) was sequenced and annotated to better identify *C.chinensis* and related species. The mitogenome is 18,680 bp in length, includes the 37 typical mitochondrial genes (13 protein-coding genes, two ribosomal RNA genes and 22 transfer RNA genes) and two control regions (total length: 3,879 bp). Mitogenome organisation, nucleotide composition and codon usage are similar to the previously sequenced *Curculio* mitogenomes. All 13 protein-coding genes use ATN or TTG as start codon and end with TAA/G or incomplete stop codons (single T-). Twenty-one transfer RNA genes have the typical clover-leaf structure, while the dihydrouridine (DHU) arm of trnS1 is missing. In *Curculio* mitogenomes, the size of the control region is highly variable. Both ML and BI analyses, based on the 13 PCGs and two rRNAs from six species of Curculioninae, strongly supported the monophyly of *Curculio*. In *Curculio*, the relationships amongst included species were inferred as ((*C.chinensis* + *Curculio*. sp.) + (*Curculiodavidi* + *Curculioelephas*)), with *C.chinensis* and *C.* sp. forming a clade (BS = 100; PP = 1).

## Introduction

The typical mitogenome of insects is a circular double-stranded DNA molecule with 15-18 kb in length, encoding 13 protein-coding genes (PCGs), two ribosomal RNA genes (rRNAs), 22 transfer RNA genes (tRNAs) and also includes a large non-coding region (control region) (Boore 1999, Cameron 2014). In insects, the mitogenome has been widely used as a molecular marker to explore population genetics, phylogeny and evolution (Fenn et al. 2008, Galtier et al. 2009, Cameron 2014).

The camellia weevil, *Curculiochinensis* (Chevrolat, 1878) is widely distributed in most of China's *Camellia* spp. (family Theaceae) producing areas (Li et al. 2015). It is one of the most serious pests of tea and causes huge economic losses (Li et al. 2015). Since different species exhibit distinct responses to specific biocontrol agents and pesticides, accurate species identification is very important in pest management (Zhou et al. 2020). However, the camellia weevil is often difficult to identify using morphological characteristics of the larvae. It is impractical to identify camellia weevil by rearing larvae to adults because the larvae are long-lived and difficult to rear when removed from the seed (Shu et al. 2013, He et al. 2014). Molecular identification has proven to be reliable and effective for species-level identification of insects at any life stage (Chen et al. 2016).

In this study, we sequenced and annotated the mitogenome of *C.chinensis* and analysed its characteristics. In addition, we reconstructed the molecular phylogenetic relationships of *C.chinensis* and other species of the genus *Curculio*. The molecular data presented here will be useful for studies on identification and evolution in *C.chinensis* and related species.

## Materials and methods

### Sample collection and DNA extraction

Adult specimens of *C.chinensis* were collected from *Camellia* spp. in Yunguanshan Forest Farm, Guiyang City, Guizhou Province, China (26.48208727°N, 106.75480714°E, July 2020) (*C.chinensis* is a host-specifc predator of the seeds of *Camellia* spp.). All fresh specimens were preserved in 100% ethyl alcohol and deposited in a -20℃ freezer at the laboratory of Guizhou Academy of Forestry, Guiyang. Identification of adult specimens was based on morphological characteristics (Chao and Chen 1980). Whole genomic DNA was extracted from thorax muscle tissues using the Biospin Insect Genomic DNA Extraction Kit (BioFlux) following the manufacturer's instructions. Voucher specimens are stored in the insect collection of Guizhou Academy of Forestry.

### Mitogenome sequencing, assembly, annotation and bioinformatic analyses

The complete mitogenome of *C.chinensis* was sequenced using NGS (next-generation sequencing) (Illumina HiSeq X10; Biomarker Technologies Corporation, Beijing, China). About 1.26 Gb clean data were assembled into a complete circular mitogenome by NOVOPlasty v.2.7.0 (Dierckxsens et al. 2016) using the COX1 sequence of *Curculiodavidi* Fairmaire, 1878 (GenBank accession: NC_034293) (Xu et al. 2017) as an initial seed. The mitogenome was annotated using MITOZ v.1.04 (Meng et al. 2019) and checked manually in Geneious v.8.1.3 (Biomatters, Auckland, New Zealand). The tRNA secondary structures were manually drawn using Adobe Illustrator CC2017, based on the MITOS Web Server (Bernt et al. 2013) predictions. The mitogenome map was drawn with the programme Organellar Genome DRAW (OGDRAW) (Lohse et al. 2013). Bioinformatic analyses, including nucleotide composition, composition skew, codon usage of PCGs, relative synonymous codon usage (RSCU) and mitogenomic organisation tables were conducted using PhyloSuite v.1.2.2 (Zhang et al. 2019).

### Molecular phylogenetic analysis

A total of six mitogenomes from two genera of Curculioninae were used for the phylogenetic analyses (Table 1). We used as much mitogenome data for the genus *Curculio* in NCBI as possible. Of these, four species belong to *Curculio* (the ingroup), while the remaining two species from the genus *Anthonomus* Germar, 1817 were chosen as outgroup. Nucleotide sequences (without stop codons) for the 13 PCGs were aligned using MAFFT v.7 (Katoh and Standley 2013) with the G-INS-i (accurate) strategy and codon alignment mode (Code table: Invertebrate mitochondrial genetic codon). The rRNAs genes (rrnL and rrnS) were aligned using MAFFT v.7 (Katoh and Standley 2013) with the Q-INS-I algorithm (which takes account of the secondary structure of rRNA genes). Ambiguously aligned areas were removed using Gblocks v.0.91b (Talavera and Castresana 2007), respectively. Gene alignments were concatenated using PhyloSuite v.1.2.2 (Zhang et al. 2019). Partitioning scheme and nucleotide substitution models for Maximum Likelihood (ML) and Bayesian Inference (BI) phylogenetic analyses were selected with PartitionFinder2 (Lanfear et al. 2017) using the Bayesian Information Criterion (BIC) (Suppl. materials 1, 2). ML analyses were reconstructed by IQ-TREE v.1.6.3 (Nguyen et al. 2015) under the ultrafast bootstrap (UFB) approximation approach (Minh et al. 2013) with 5,000 replicates. BI analysis was performed using MrBayes v.3.2.7a (Ronquist et al. 2012) in the CIPRES Science Gateway (Miller et al. 2010) with four chains (one cold chain and three hot chains). Two independent runs of 2,000,000 generations were carried out with sampling every 1,000 generations. The first 25% of trees were discarded as burn-in. After the average standard deviation of split frequencies fell below 0.01, stationarity was assumed.

## Results and discussion

### Mitogenome organisation and nucleotide composition

**Figure 1.**
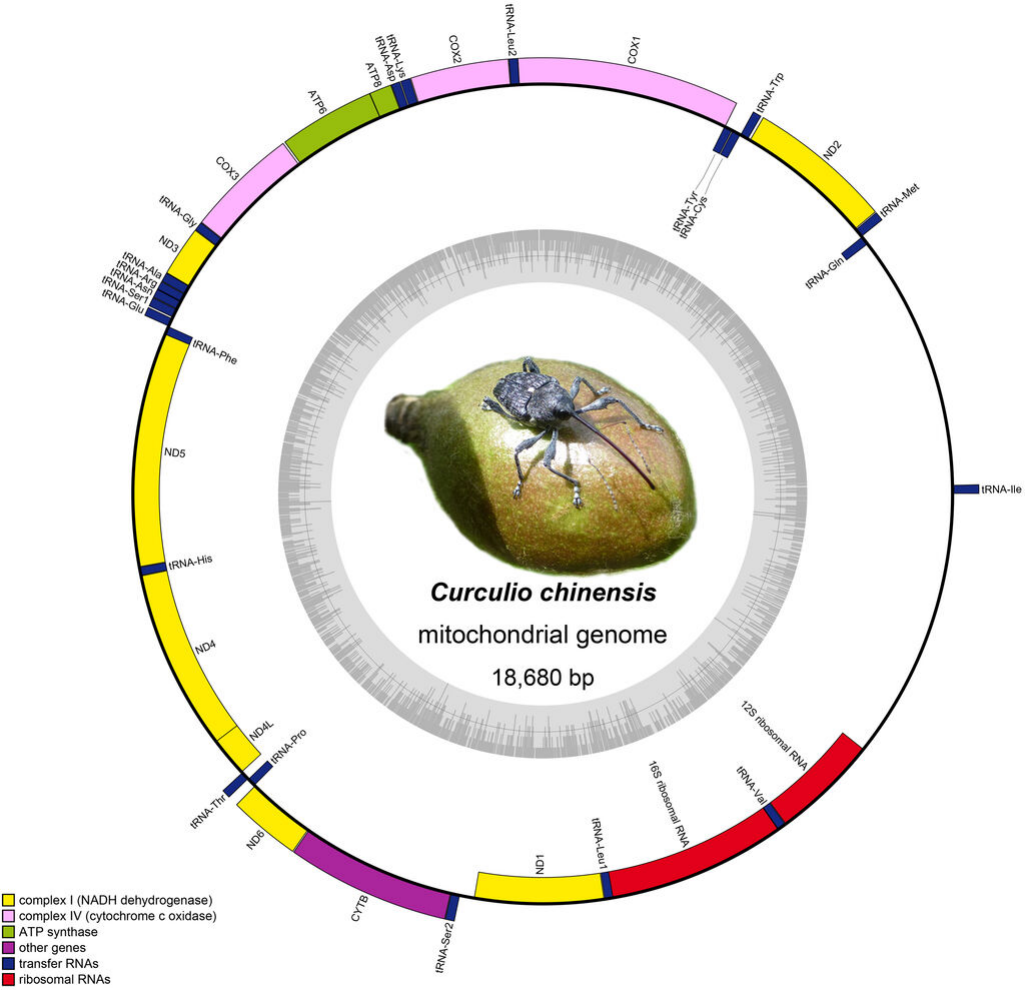
Circular map of the mitogenome of *C.chinensis*. The outer circle shows the gene map of *C.chinensis* and the genes outside the map are coded on the major strand (J-strand), whereas the genes on the inside of the map are coded on the minor strand (N-strand). Genes are represented by different colour blocks.

**Figure 2.**
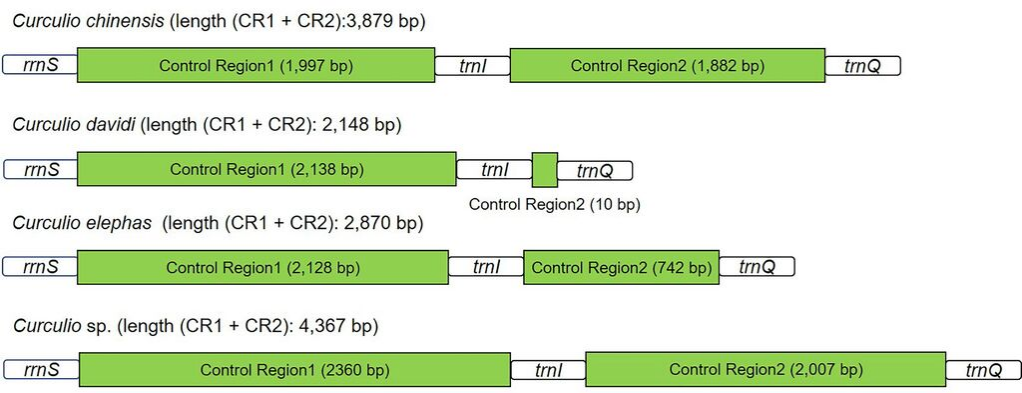
Control regions in the four complete *Curculio* mitogenomes.

The mitogenome of *C.chinensis* is a double-stranded circular DNA molecule, containing 37 typical mitochondrial genes (13 PCGs, 22 tRNAs and two rRNAs) and two control regions (Table 2, Fig. 1), which are common in Curculioninae mitogenomes (Narakusumo et al. 2020). The newly-sequenced mitogenome (length: 18,680 bp) is medium-sized compared to other *Curculio* mitogenomes (ranging from 16,852 bp *Curculiodavidi*, GenBank accession: NC_034293 to 19,216 bp *Curculio* sp., GenBank accession: MG728095) (Xu et al. 2017). Variation in the size of the control region is the main source of the length variation in *Curculio* mitogenomes (Fig. 2). The mitogenome of *C.chinensis* has the same gene order as other previously sequenced *Curculio* species (Xu et al. 2017). A total of 71 overlapping nucleotides were found in ten pairs of neighbouring genes, the longest overlap (23 bp) being identified between the trnL1 and rrnL. Furthermore, there are 151 intergenic nucleotides dispersed across 13 gene boundaries and the longest intergenic region (103 bp) is located between trnS2 and nad1.

The nucleotide content of the *Curculio* mitogenomes exhibit strong AT bias: 76.9%-77.5% in the whole genome, 75.7%-76.1% in the PCGs, 76.8%-78.3% in the tRNAs, 76.9%-78.8% in the rRNAs and 78.8%-83.7% in the control region (Table 3). In every sequenced mitogenome of *Curculio*, PCGs have the lowest AT content, while the control region has the highest AT content (Table 3). All four *Curculio* mitogenomes have positive AT-skews (0.052–0.062) and negative GC-skews (−0.203 to −0.17), similar to other recently reported weevil mitogenomes (Apriyanto and Tambunan 2020, Song et al. 2020, Wang et al. 2020, Wang et al. 2021) and most other insects (Wei et al. 2010).

### Protein-coding genes

**Figure 3.**
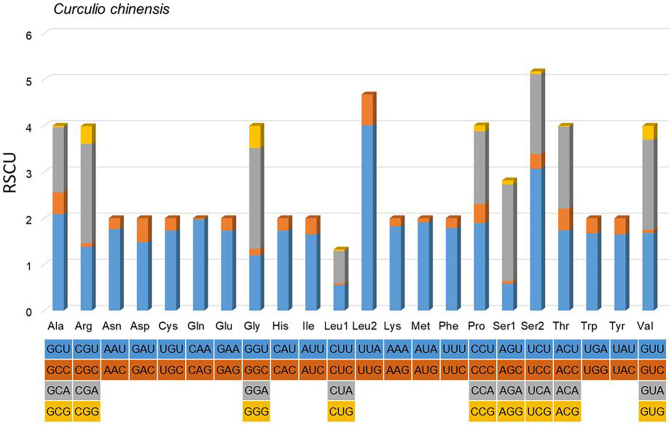
Relative synonymous codon usage (RSCU) of the mitogenome of *C.chinensis*. The stop codon is not shown.

The total size of all 13 PCGs of *C.chinensis* is 11,160 bp, accounting for 59.74% of the entire mitogenome (Table 3). In 13 PCGs, nad2, cox1, cox2, atp8, atp6, cox3, nad3, nad5, nad4, nad4L, nad6 and cob use ATN (ATA/T/G/C) as start codon, while nad1 is initiated by TTG, which is common for *Curculio* mitogenomes (Xu et al. 2017). All PCGs stopped with TAA/G or their incomplete form single T-. The incomplete termination codon single T- can be completed by post-transcriptional polyadenylation (Ojala et al. 1981). The AT-skews of all PCGs amongst *Curculio* range from -0.146 (*C.davidi*) (Xu et al. 2017) to -0.133 (*C.chinensis* and *Curculio* sp.), showing a biased use for the T nucleotide. The relative synonymous codon usage (RSCU) of *C.chinensis* mitogenome is presented in Fig. 3, indicating Leu, Phe and Ile are the three most frequently used amino acids. In the new mitogenome, the four most frequently utilised codons are UUA-Leu, UUU-Phe, AUU-Ile and AUA-Met. The most frequently used codons are composed of A nucleotide or U nucleotide, which reflects the high AT content of PCGs.

### Transfer and ribosomal RNA genes

**Figure 4.**
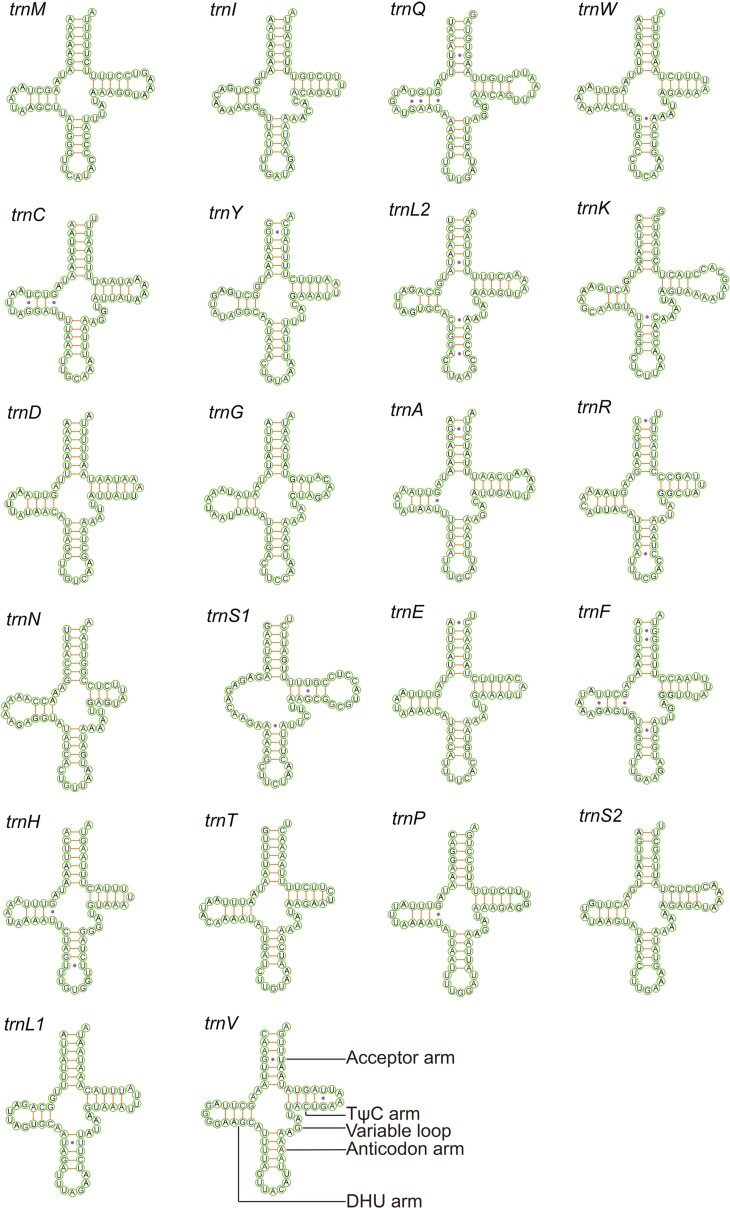
Secondary structures of 22 tRNAs in the mitogenome of *C.chinensis*. Lines (-) indicate Watson-Crick base pairings, whereas dots (·) indicate unmatched base pairings.

The typical sets of 22 tRNAs were identified with the size ranging from 62 bp (trnR) to 71 bp (trnK) (Table 2). The AT content of tRNAs (76.8%-78.3%) was slightly higher than that of the PCGs (75.7%-76.1%) (Table 3). Most tRNAs have clover-leaf secondary structures, except for trnS1, where the dihydrouridine (DHU) arm became a simple loop (Fig. 4). This feature is common in metazoan mitogenomes (Garey and Wolstenholme 1989). A total of 30 mismatched base pairs belonging to six types (U-G, U-U, A-C, A-G, U-C and A-A) were found in the arm structures of the 22 tRNAs.

The length of rrnS and rrnL genes ranges from 2,059 bp (*C.* sp.) to 2,152 bp (*C.chinensis*) and AT content of rRNAs is conserved in the *Curculio* (Table 3). For *C.chinensis*, the rrnL gene (length: 1329 bp) is encoded between trnL1 and trnV and the rrnS gene (length: 788 bp) is encoded between trnV and the control region, similar to other sequenced *Curculio* (Xu et al. 2017).

### Control region

The control region regulates the replication and transcription of mtDNA (Boore 1999, Cameron 2014). In each sequenced *Curculio* mitogenome, the control region is subdivided by trnI into two parts (control region1 and control region2) (Fig. 2). Control region1 (CR1) is located between rrnS and trnI, while control region2 (CR2) is located between trnI and trnQ. The length and AT content of CR1 (1,997-2,360 bp and 83%-85.6%) are slightly higher than CR2 (10-2,007 bp and 60%-90%) (Table 3).

### Phylogenetic relationships

**Figure 5.**
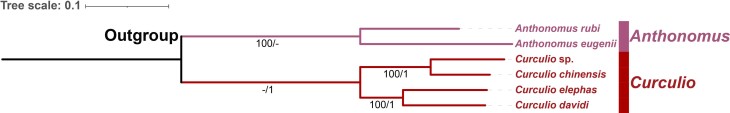
ML and BI phylogenetic trees for *Curculio*, based on the nucleotide sequence data of 13 PCGs and two rRNAs from *C.chinensis* and other five species belonging to two related genera of Curculioninae. Bootstrap support values (BS) and Bayesian posterior probabilities (PP) are indicated on the branch.

Based on ML and BI analyses of nucleotide data of 13 PCGs and two rRNAs, we reconstructed the phylogenetic relationships of four species of *Curculio*. The trees of both analyses have congruent topologies, with all branches strongly supported (Fig. 5). Furthermore, relationships recovered in our analyses are similar to those found by Song et al. (Song et al. 2020), but we only focused on the phylogenetic relationships within *Curculio*. The monophyly of the genus *Curculio* was recovered with strong support, consistent with the previous study (Song et al. 2020). In *Curculio*, the relationships amongst included species were inferred as ((*C.chinensis* + *C.* sp.) + (*Curculiodavidi* + *Curculioelephas* Fabricius, 1781)), with *C.chinensis* and *C.* sp. forming a clade. In China's *Camellia* spp. producing areas, both *C.chinensis* and *C.* sp. are host-specific predators of the seeds of *Camellia* spp. The topologies of the phylogenetic trees reconstructed by us strongly supported the sister relationship between these two *Curculio* species (BS = 100; PP = 1), which may reflect a convergent evolutionary phenomenon in *Curculio* species with *Camellia* spp. as their host.

## Supplementary Material

D126F915-3DD1-5185-AF08-1613E4C31AD3 10.3897/BDJ.9.e69196.suppl1 Supplementary material 1 Table S1 Data type docx Brief description The best partitioning schemes and substitution models for PCG123 + rRNA dataset comprising 13 PCGs and two rRNAs of six species of Curculioninae used for ML phylogenetic analyses. File: oo_592857.docx https://binary.pensoft.net/file/592857 Kai Hu, Zaihua Yang

5696AECB-EE06-5065-AA92-FDD8201B7E98 10.3897/BDJ.9.e69196.suppl2 Supplementary material 2 Table S2 Data type docx Brief description The best partitioning schemes and substitution models for PCG123 + rRNA dataset comprising 13 PCGs and two rRNAs of six species of Curculioninae used for BI phylogenetic analyses. File: oo_592858.docx https://binary.pensoft.net/file/592858 Kai Hu, Zaihua Yang

## Figures and Tables

**Table 1.** Mitogenomes of the six Curculioninae taxa used in this study.

Subfamily	Species	Accession number	Reference
Curculioninae	*Anthonomuseugenii*	NC_044711	*van de Vossenberg et al. 2019*
	*Anthonomusrubi*	NC_044714	*van de Vossenberg et al. 2019*
	*Curculiochinensis*	MZ417388	This study
	*Curculio* sp.	MG728095	Unpublished
	*Curculiodavidi*	NC_034293	Xu et al. 2017
	*Curculioelephas*	KX087269	Unpublished

**Table 2.** Mitogenomic organisation of *C.chinensis*.

Gene name	Location		Size (bp)	Intergenic nucleotides	Codon		Strand
	From	To			Start	Stop	
trnI	1	65	65				+
CR2	66	1947	1882				+
trnQ	1948	2016	69				-
trnM	2018	2085	68	1			+
nad2	2089	3096	1008	3	ATA	TAA	+
trnW	3111	3174	64	14			+
trnC	3174	3239	66	-1			-
trnY	3242	3305	64	2			-
cox1	3298	4842	1545	-8	ATT	TAA	+
trnL2	4838	4902	65	-5			+
cox2	4903	5586	684		ATT	TAA	+
trnK	5588	5658	71	1			+
trnD	5661	5725	65	2			+
atp8	5726	5884	159		ATT	TAA	+
atp6	5881	6552	672	-4	ATA	TAA	+
cox3	6563	7343	781	10	ATT	T	+
trnG	7344	7407	64				+
nad3	7408	7761	354		ATT	TAG	+
trnA	7760	7826	67	-2			+
trnR	7827	7888	62				+
trnN	7887	7950	64	-2			+
trnS1	7951	8017	67				+
trnE	8025	8088	64	7			+
trnF	8089	8153	65				-
nad5	8137	9873	1737	-17	ATT	TAA	-
trnH	9874	9936	63				-
nad4	9937	11272	1336		ATG	T	-
nad4L	11266	11559	294	-7	ATG	TAA	-
trnT	11562	11626	65	2			+
trnP	11627	11692	66				-
nad6	11695	12198	504	2	ATT	TAA	+
cob	12202	13338	1137	3	ATA	TAA	+
trnS2	13339	13405	67				+
nad1	13509	14459	951	103	TTG	TAG	-
trnL1	14461	14525	65	1			-
rrnL	14503	15831	1329	-23			-
trnV	15830	15895	66	-2			-
rrnS	15896	16683	788				-
CR1	16684	18680	1997				+

**Table 3.** Base composition and skewness of mitogenomes of *Curculiochinensis*, *Curculio* sp., *Curculiodavidi* and *Curculioelephas*.

Feature	Length	A+T%	AT-skew	GC-skew
*C.chinensis*, *C.* sp., *C.davidi* and *C.elephas*				
Whole genome	18680/19216/16852/17591	76.9/77/77.2/77.5	0.056/0.06/0.062/0.052	-0.19/-0.203/-0.185/-0.17
PCGs	11160/11091/11154/10989	76/75.7/75.9/76.1	-0.133/-0.133/-0.146/-0.145	-0.038/-0.043/-0.049/-0.055
tRNAs	1442/1444/1440/1447	76.8/77.8/76.8/78.3	0.036/0.02/0.011/0.026	0.12/0.121/0.12/0.144
rRNAs	2117/2059/2152/2084	78.8/78.3/76.9/78.3	-0.064/-0.086/-0.062/-0.063	0.345/0.348/0.315/0.307
CR1	1997/2360/2138/2128	84.2/83/83.7/85.6	-0.014/0.058/0.089/0.058	-0.478/-0.606/-0.24/-0.24
CR2	1882/2007/10/742	71.4/73.9/90/69	-0.023/-0.076/-0.333/-0.011	0.235/0.179/-1/0.139
Control region (CR1 + CR2)	3879/4367/2148/2870	78/78.8/83.7/81.3	-0.018/0/0.087/0.043	-0.028/-0.161/0.234/-0.078
