# Supplementary material for: The complete mitogenome of Curculiochinensis (Chevrolat, 1878) (Coleoptera: Curculionidae: Curculioninae)
Source: Biodivers Data J. 2021 Oct 25;9:e69196. doi: 10.3897/BDJ.9.e69196 (PMC8560736; doi:10.3897/BDJ.9.e69196)
Supplement: Supplementary material 2 — Table S2 [file bdj-09-e69196-s002.docx]

**Table S2.** The best partitioning schemes and substitution models for PCG123 + rRNA dataset comprising 13 PCGs and two rRNAs of six species of Curculioninae used for BI phylogenetic analyses.

| Optimal partition | Model | Subset partition |
| --- | --- | --- |
| Partition1 | GTR+I+G | *nad3*, *atp6*, *cox1*, *cox2*, *cytb*, *cox3* |
| Partition2 | GTR+I | *atp8*, *nad2*, *nad6* |
| Partition3 | GTR+I+G | *nad5*, *nad4L*, *nad4*, *nad1* |
| Partition4 | GTR+G | *rrnS*, *rrnL* |
